# Undernutrition Disrupts Cecal Microbiota and Epithelium Interactions, Epithelial Metabolism, and Immune Responses in a Pregnant Sheep Model

**DOI:** 10.1128/spectrum.05320-22

**Published:** 2023-03-28

**Authors:** Weibin Wu, Huizhen Lu, Jianbo Cheng, Zhaoyu Geng, Shengyong Mao, Yanfeng Xue

**Affiliations:** a College of Animal Science and Technology, Anhui Agricultural University, Hefei, China; b Biotechnology Center, Anhui Agricultural University, Hefei, China; c College of Animal Science and Technology, Nanjing Agricultural University, Nanjing, China; Jilin University

**Keywords:** cecal microbiota, epithelial metabolism, fermentation parameters, immune response, signaling pathway

## Abstract

Undernutrition may change cecal microbiota-epithelium interactions to influence cecal feed fermentation, nutrient absorption and metabolism, and immune function. Sixteen late-gestation Hu-sheep were randomly divided into control (normal feeding) and treatment (feed restriction) groups to establish an undernourished sheep model. Cecal digesta and epithelium were collected to analyze microbiota-host interactions based on 16S rRNA gene and transcriptome sequencing. Results showed that cecal weight and pH were decreased, volatile fatty acids and microbial proteins concentrations were increased, and epithelial morphology was changed upon undernutrition. Undernutrition reduced the diversity, richness, and evenness of cecal microbiota. The relative abundances of cecal genera involved in acetate production (*Rikenellaceae* dgA-11 gut group, *Rikenellaceae* RC9 gut group, and *Ruminococcus*) and negatively correlated with butyrate proportion (*Clostridia* vadinBB60 group_norank) were decreased, while genera related to butyrate (*Oscillospiraceae*_uncultured and *Peptococcaceae*_uncultured) and valerate (*Peptococcaceae*_uncultured) production were increased in undernourished ewes. These findings were consistent with the decreased molar proportion of acetate and the increased molar proportions of butyrate and valerate. Undernutrition changed the overall transcriptional profile and substance transport and metabolism in cecal epithelium. Undernutrition suppressed extracellular matrix-receptor interaction and intracellular phosphatidyl inositol 3-kinase (PI3K) signaling pathway then disrupted biological processes in cecal epithelium. Moreover, undernutrition repressed phagosome antigen processing and presentation, cytokine-cytokine receptor interaction, and intestinal immune network. In conclusion, undernutrition affected cecal microbial diversity and composition and fermentation parameters, inhibited extracellular matrix-receptor interaction and the PI3K signaling pathway, and then disrupted epithelial proliferation and renewal and intestinal immune functions. Our findings exposed cecal microbiota-host interactions upon undernutrition and contribute to their further exploration.

**IMPORTANCE** Undernutrition is commonly encountered in ruminant production, especially during pregnancy and lactation in females. Undernutrition not only induces metabolic diseases and threatens pregnant mothers’ health, but also inhibits fetal growth and development, leading to weakness or even death of fetuses. Cecum works importantly in hindgut fermentation, providing volatile fatty acids and microbial proteins to the organism. Intestinal epithelial tissue plays a role in nutrient absorption and transport, barrier function, and immune function. However, little is known about cecal microbiota and epithelium interactions upon undernutrition. Our findings showed that undernutrition affected bacterial structures and functions, which changed fermentation parameters and energy regimens, and therefore affected the substance transport and metabolism in cecal epithelium. Extracellular matrix-receptor interactions were inhibited, which repressed cecal epithelial morphology and cecal weight via the PI3K signaling pathway and lowered immune response function upon undernutrition. These findings will help in further exploring microbe-host interactions.

## INTRODUCTION

Malnutrition in ruminant production is commonly due to feed fluctuations caused by seasonal changes or cost controls. The susceptibility to malnutrition of ruminants in pregnancy and lactation is likely to increase because of the high energy requirements in these specific physiological periods. Since about 80% of the fetal birth weight is formed during late gestation (1), ewes' energy requirements for singleton and twin pregnancies are 150% and 200% higher than usual, respectively (2). However, the gradually increased fetal weight and uterus size squeeze the rumen, leading to reduced rumen volume and decreased feed intake. In particular, ewes with multiple lambs will have a more increased energy demand and more reduced feed intake than ewes with single lambs, resulting in more severe undernutrition during late gestation (2). Undernutrition will not only induce pregnancy toxemia and a threat to maternal health but also inhibit fetal growth and development and lead to weak or even dead lambs (3).

The gut microbiota acts as a signaling hub which links external factors, such as diet, to the body's immune signals and metabolic processes, influencing host immune responses and metabolic homeostasis (4). There is evidence that malnutrition affects the gastrointestinal microbiota structure and barrier function (5). Undernutrition changes microbial community composition and fermentation parameters in the colon of Hu-sheep (6). Undernutrition reprograms the cross talk between ruminal microbiota and epithelium and alters nutrients metabolic processes in the ruminal epithelium of Hu-sheep (7). Undernutrition also alters intestinal and systemic immune homeostasis by affecting the composition of intestinal microbiota (8). Microbiota in the hindgut is critical to the health and efficient production by ruminants. Cecum plays a vital role in hindgut fermentation, which ferments about 17% of the digestible cellulose and provides 12% of the total volatile fatty acids (VFAs) to the organism (9). VFAs account for up to 75% of the energy supply for ruminants (10). Previous studies have shown that a high-grain diet disrupts the balance of cecum microbiota and leads to cecal mucosal damage in ruminants (9). Restricted feeding also affects cecal microbiota and alters cecal weight in monogastric animals, such as the hen (11) and rat (12). However, although the cecum is not negligible in the hindgut of ruminants, very little is known about the changes that occur in the cecum during malnutrition. Given that the volume, turnover rate, and fermentation in the rumen of Hu-sheep are affected by undernutrition (7), the fermentation in the cecum must be changed. Here, we hypothesized that malnutrition for sheep during late gestation would affect the composition and function of cecal microbiota, thereby influencing the nutrient metabolism and immune response in cecal epithelium.

Therefore, this study aimed to investigate the interaction of cecal microbiota and epithelium in response to malnutrition through 16S rRNA gene sequencing of cecal digesta and transcriptome sequencing of cecal epithelium.

## RESULTS

### Undernutrition affected cecal fermentation mode and epithelial morphology.

**FIG 1:**
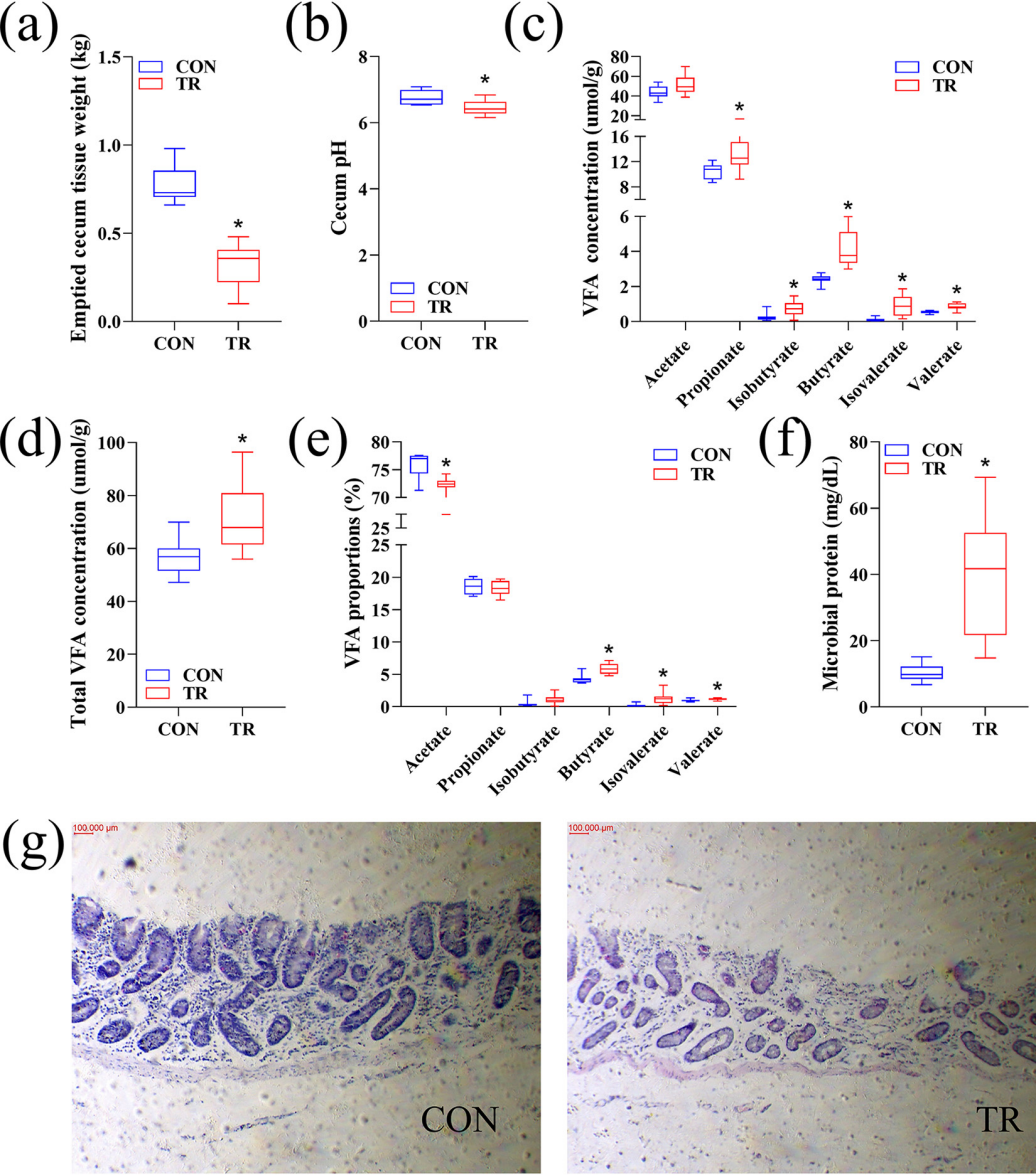
Undernutrition affected the cecal weight, fermentation parameters, and morphology. (a) Emptied cecal weight in the CON and TR groups. (b) Cecal pH. (c) Cecal VFA concentrations. (d) Cecal total VFA concentration. (e) Molar proportions of VFAs. (f) Cecal microbial protein. (g) Hematoxylin and eosin-stained sections of cecal epithelium. Asterisks indicate significant differences (*P* < 0.05).

From the 115th day of gestation, ewes in the control (CON) and treated (TR) groups were fed 100% and 30% of the *ad libitum* feeding level, respectively, for 15 days to establish an undernourished sheep model. Blood β-hydroxybutyric acid (BHBA) concentration of the TR group was 5.6 times higher (*P* < 0. 001) than in the CON group and was above the threshold (0.8 mmol/liter), while blood glucose concentration of the TR group was just 63% (*P* < 0. 001) of the CON group and was below the threshold (3 mmol/liter) (13), which indicated the successful establishment of the undernourished sheep model during late gestation. The weight of empty cecum (*P* < 0.001) and cecal pH (*P* = 0.02) in the TR group were lower than those of the CON group (Fig. 1a and b). Compared to the CON group, undernutrition significantly increased the concentrations of propionate (*P* = 0.026), isobutyrate (*P* = 0.018), butyrate (*P* < 0.001), isovalerate (*P* = 0.002), valerate (*P* = 0.001), and total VFAs (*P* = 0.029) in the cecal digesta (Fig. 1c and d). Further, the molar ratios of butyrate (*P* = 0.001), isovalerate (*P* = 0.006), and valerate (*P* = 0.023) were increased, while the molar ratio of acetate (*P* = 0.002) was decreased and the molar ratios of propionate (*P* = 0.964) and isobutyrate (*P* = 0.088) remained unchanged in the cecal digesta of TR ewes compared with ratios in the CON ewes (Fig. 1e). The microbial protein content in the cecal digesta of the TR group was significantly higher (*P* = 0.001) than that of the CON group (Fig. 1f). In addition, a significant detachment of the lamina propria of the cecal epithelium occurred in the TR group compared to the CON group (Fig. 1g).

### Undernutrition changed cecal microbiota diversity and composition.

**FIG 2:**
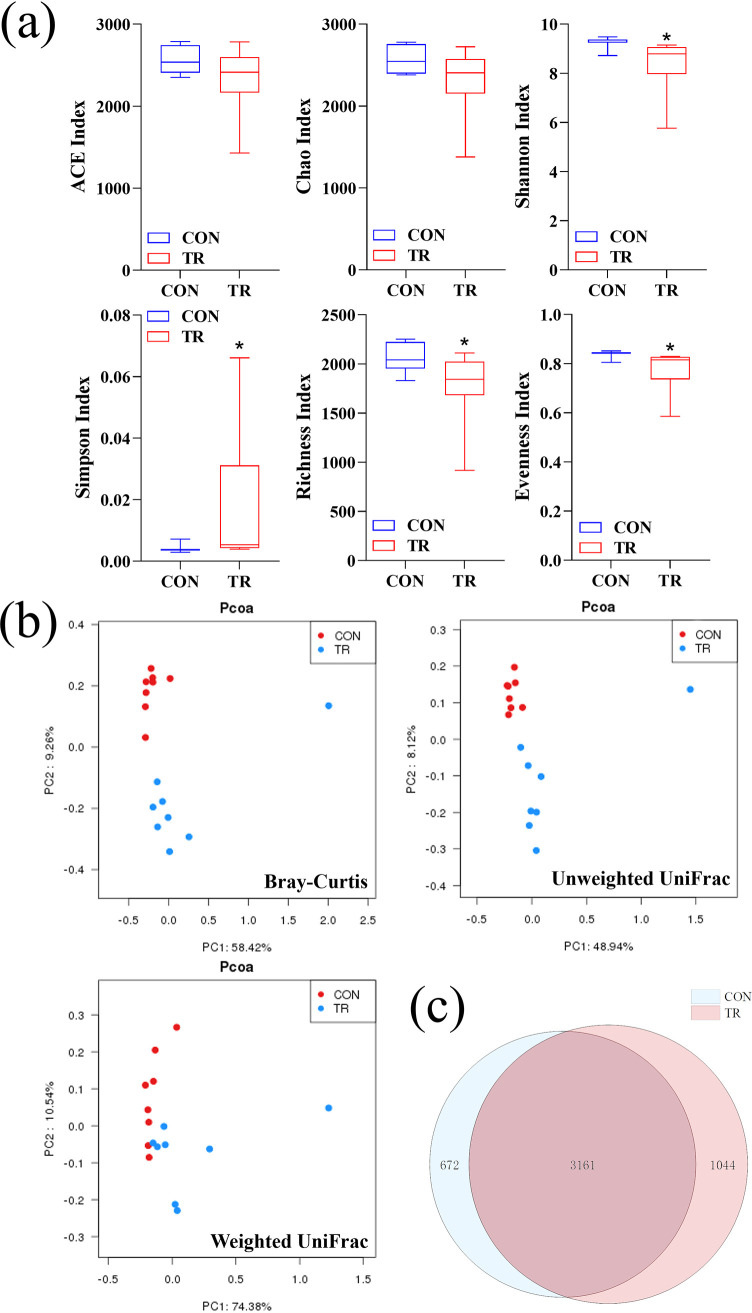
Effect of undernutrition on the diversity of bacterial communities in cecal digesta. (a) Alpha diversity index. (b) PCoA plots (Bray-Curtis, unweighted UniFrac, and weighted UniFrac) of bacterial communities, based on OTUs. (c) Venn diagram of OTUs in two groups. Asterisks indicate significant differences (*P* < 0.05).

To explore how undernutrition changed cecal fermentation, we extracted DNA from cecal digesta and performed 16S rRNA gene sequencing to study the alteration of cecal microbiota. Rarefaction curves of all samples converged to a plateau, indicating sufficient sequencing depth (see Fig. S1 in the supplemental material). Compared to the CON group, Shannon (*P* = 0.003), richness (*P* = 0.036), and evenness (*P* = 0.005) diversity indices decreased significantly, while the Simpson index (*P* = 0.006) increased significantly and the abundance-based coverage estimator (*P* = 0.141) and Chao (*P* = 0.115) indices remained unchanged in the TR group (Fig. 2a). Principal-coordinate analysis (PCoA) based on Bray-Curtis, unweighted UniFrac, and weighted UniFrac metrics demonstrated the differences of the cecal microbial community between the CON and TR groups (Fig. 2b). A Venn diagram also illustrated that only 3,161 operational taxonomic units (OTUs) were identified in the two groups, while 672 and 1,044 unique OTUs were distributed in the CON and TR groups, respectively (Fig. 2c).

**FIG 3:**
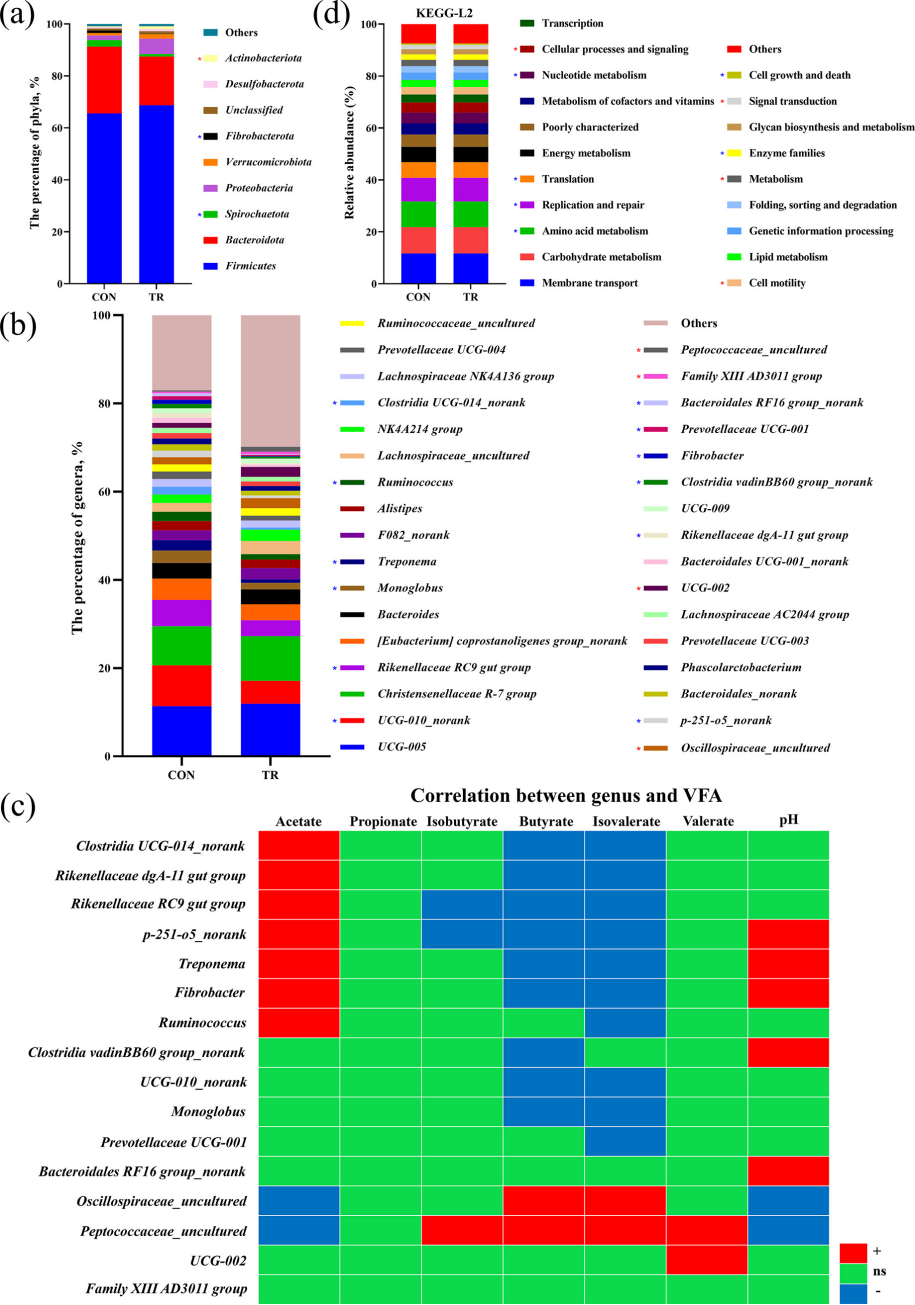
Undernutrition changed the structures and functions of bacterial communities in cecal digesta. (a) Relative abundances of bacterial communities at the phylum level. (b) Relative abundances of bacterial communities at the genus level. (c) Correlations between the changed bacterial communities and fermentation parameters in the cecal digesta. +, significantly positive correlation (*P* < 0.05); −, significantly negative correlation (*P* < 0.05); ns, nonsignificant correlation (*P* > 0.05). (d) Abundance distribution map of cecal microbiota based on KEGG prediction function. Red asterisks indicate significant increases (*P* < 0.05) and blue asterisks indicate significant decreases (*P* < 0.05) of abundances distributed in the indicated biological process.

Distinct characteristics were presented in the relative abundances of cecal bacteria at the phylum and genus levels, with each taxon representing more than 0.5% in at least one group. In detail, 36 phyla were identified in both groups; *Firmicutes*, *Bacteroidota*, *Spirochaetota*, *Proteobacteria*, and *Verrucomicrobiota* were the dominant phyla (relative abundance, >1%) in the CON group, while *Firmicutes*, *Bacteroidota*, *Proteobacteria*, *Verrucomicrobiota*, and *Actinobacteriota* were the dominant phyla in the TR group. At the phylum level, the relative abundances of *Spirochaetes* and *Fibrobacterota* decreased (*P* < 0.05), while that of *Actinobacteria* increased (*P* < 0.05) in the TR group (Fig. 3a). A total of 44 genera were identified with relative abundances of more than 0.5% in at least one group. At the genus level, the relative abundances of UCG-010_norank, *Rikenellaceae* RC9 gut group, *Monoglobus*, Treponema, *Ruminococcus*, *Clostridia* UCG-014_norank, p-251-o5_norank, *Rikenellaceae* dgA-11 gut group, *Clostridia* vadinBB60 group_norank, *Fibrobacter*, *Prevotellaceae* UCG-001, and *Bacteroidales* RF16 group_norank were reduced (*P* < 0.05), while those of *Oscillospiraceae*_uncultured, UCG-002, Family XIII AD3011 group, and *Peptococcaceae*_uncultured were increased (*P* < 0.05) in the TR group (Fig. 3b).

To explore the association between cecal microbiota and fermentation parameters, we evaluated the correlation between significantly altered bacterial genera and VFA molar proportions and pH. The relative abundances of *Oscillospiraceae*_uncultured and *Peptococcaceae*_uncultured were mainly negatively correlated with the molar ratio of acetate and positively correlated with the molar ratios of butyrate, isovalerate, and valerate. The relative abundances of *Clostridia* UCG-014_norank, *Rikenellaceae* dgA-11 gut group, *Rikenellaceae* RC9 gut group, p-251-o5_norank, Treponema, *Fibrobacter*, and *Ruminococcus* were mainly negatively correlated with the molar ratios of butyrate and isovalerate and positively correlated with the molar ratio of acetate. The molar ratio of valerate in the cecum was positively correlated with *Peptococcaceae*_uncultured and UCG-002 only. Cecal pH was positively correlated with the relative abundances of p-251-o5_norank, Treponema, *Fibrobacter*, *Clostridia* vadinBB60 group_norank, and *Bacteroidales* RF16 group_norank and negatively correlated with those of *Oscillospiraceae*_uncultured and *Peptococcaceae*_uncultured (Fig. 3c). Functional predication analysis of cecal bacteria at the second level of the Kyoto Encyclopedia of Genes and Genomes (KEGG) database showed that cellular processes and signaling, cell motility, metabolism, and signal transduction were enhanced, while amino acid metabolism, replication and repair, translation, nucleotide metabolism, enzyme families, and cell growth and death were weakened in the TR group compared to the CON group (Fig. 3d).

### Undernutrition reprogrammed nutrient transport, metabolism, and the energy system.

**FIG 4:**
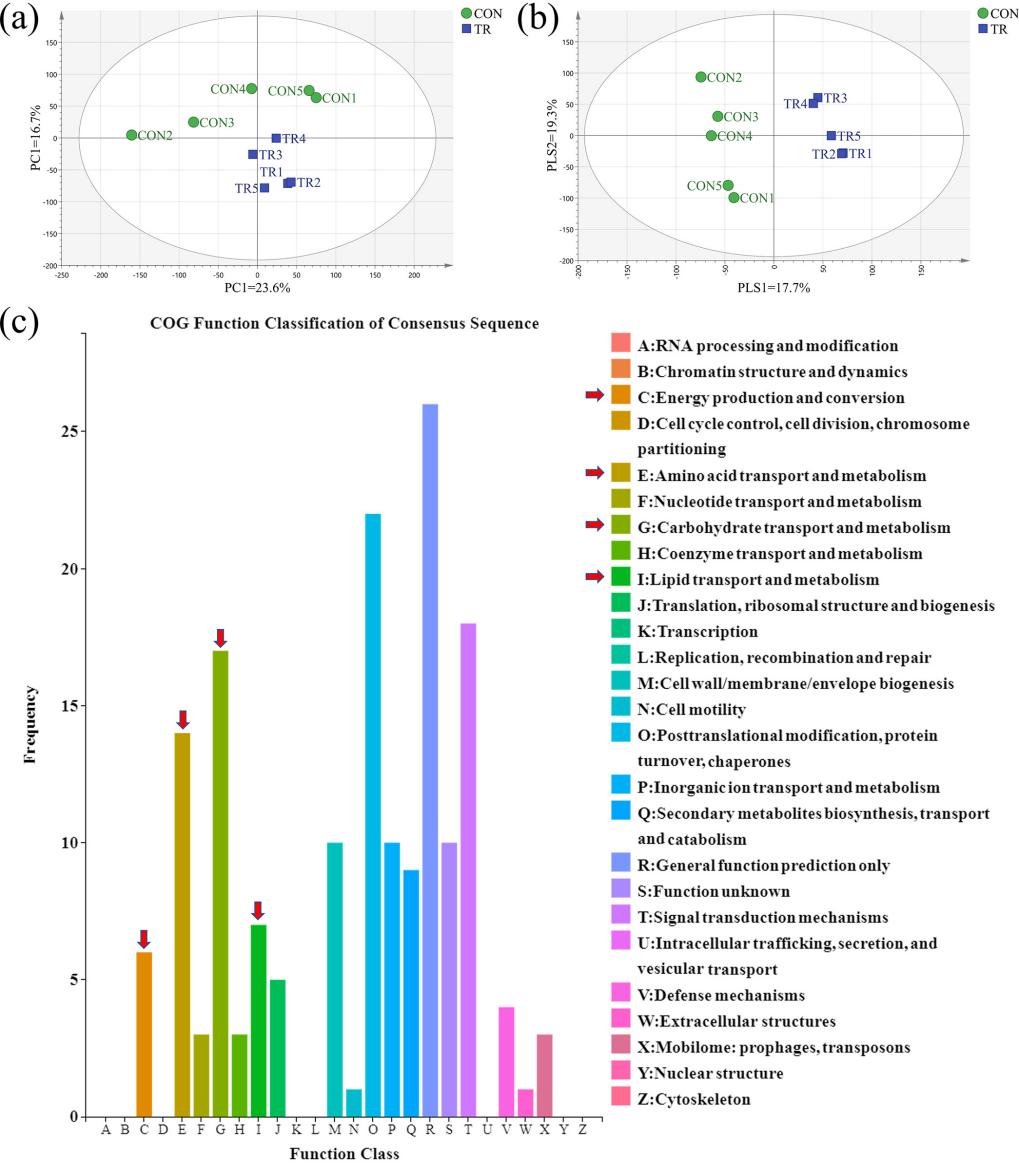
Effect of undernutrition on the transcriptome profiles of cecal epithelium. (a) PCA of total genes in the cecal epithelium. (b) Partial least-squares discriminant analysis (PLS-DA) of total genes in the cecal epithelium [predictive ability parameter (Q^2^ cum) of 0.723, goodness-of-fit parameter (*R*^2^) of *y* = 0.993]. (c) COG function classification of DEGs in the cecal epithelium. The *x* axis indicates the function classification of COG and the *y* axis indicates the number of DEGs.

Transcriptome sequencing of cecal epithelium was used to investigate the effect of undernutrition on substrate metabolism, biological processes, signal transduction, and epithelial functions. First, both principal-component analysis and partial least-squares discriminant analysis plots showed the significant differences of total transcriptome profiles between the CON and TR groups (Fig. 4a and b). Volcano plots demonstrated that 740 differentially expressed genes (DEGs) were identified between the two groups, of which 202 DEGs were upregulated and 538 DEGs were downregulated in the TR group compared to the CON group (Fig. S2). The clustering heat map of DEG expression showed that the CON and TR groups clustered into two classes (Fig. S3). The results of real-time quantitative PCR of selected DEGs also further validated the findings of the transcriptome analysis (Fig. S4). To further analyze the functions of the DEGs between the CON and TR groups, a clusters of orthologous groups (COG) functional classification was performed. Results showed that energy production and conversion, amino acid transport and metabolism, carbohydrate transport and metabolism, and lipid transport and metabolism were enriched by DEGs in addition to the general function prediction only and signal transduction mechanisms (Fig. 4c).

**FIG 5:**
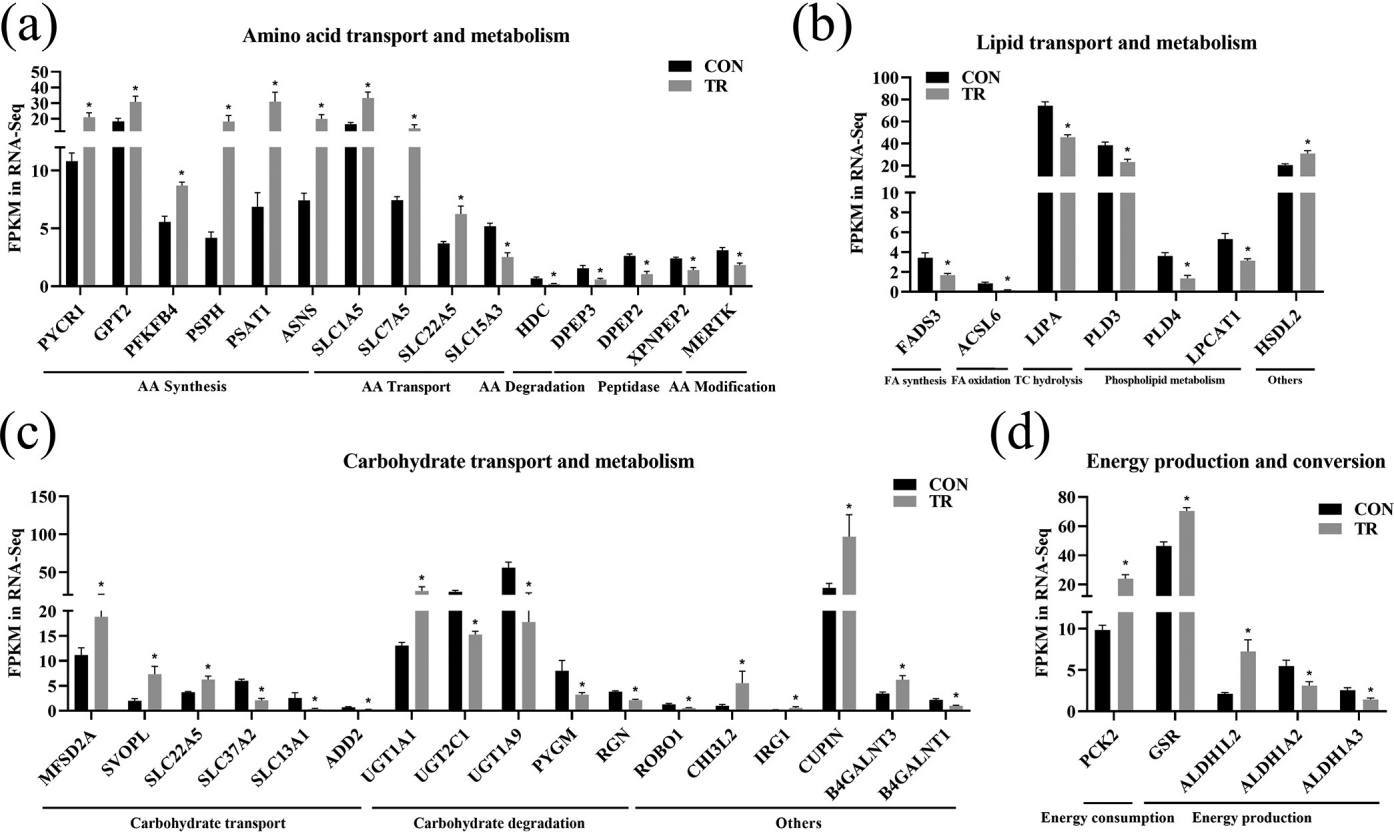
Effect of undernutrition on substance transport and metabolism in cecal epithelium. (a) Expression of genes related to amino acid transport and metabolism. (b) Expression of genes related to lipid transport and metabolism. (c) Expression of genes related to carbohydrate transport and metabolism. (d) Expression of genes related to energy production and conversion. Asterisks indicate significant differences (*P* < 0.05). AA, amino acid; FA, fatty acid; TC, total cholesterol. PYCR1, pyrroline-5-carboxylate reductase 1; GPT2, glutamic-pyruvic transaminase 2; PFKFB4, fructose-2,6-biphosphatase 4; PSPH, phosphoserine phosphatase; PSAT1, phosphoserine aminotransferase 1; ASNS, asparagine synthetase; SLC1A5, solute carrier family 1 member 5; HDC, histidine decarboxylase; DPEP3, dipeptidase 3; XPNPEP2, X-prolyl aminopeptidase 2; MERTK, MER proto-oncogene, tyrosine kinase; FADS3, fatty acid desaturase 3; ACSL6, acyl-coenzyme A synthetase long chain family member 6; LIPA, lipase A; PLD3, phospholipase D family member 3; LPCAT1, lysophosphatidylcholine acyltransferase 1; HSDL2, hydroxysteroid dehydrogenase like 2; MFSD2A, MFSD2 lysolipid transporter A lysophospholipid; SVOPL, SVOP like; ADD2, adducin 2; UGT1A1, UDP glucuronosyltransferase family 1 member A1; PYGM, glycogen phosphorylase, muscle associated; RGN, regucalcin; ROBO1, roundabout guidance receptor 1; CHI3L2, chitinase 3 like 2; IRG1, immune-responsive gene 1 protein homolog; CUPIN, cupin superfamily member 1; B4GALNT3, beta-1,4-*N*-acetyl-galactosaminyltransferase 3; PCK2, phosphoenolpyruvate carboxykinase 2; GSR, glutathione-disulfide reductase; ALDH1L2, aldehyde dehydrogenase 1 family member L2.

We further identified the DEGs associated with amino acid transport and metabolism. The results showed that DEGs related to amino acid synthesis, including *PYCR1*, *GPT2*, *PFKFB4*, *PSPH*, *PSAT1*, and *ASNS*, were all upregulated, while DEGs related to amino acid degradation (*HDC*) and modification (*MERTK*) and to peptidases (*DPEP3*, *DPEP2*, and *XPNPEP2*) were all downregulated in the TR group compared to the CON group. DEGs related to amino transport, including *SLC1A5*, *SLC7A5*, and *SLC22A5*, were all upregulated in the TR group, except for *SLC15A3* (Fig. 5a). Human and pig are deficient in many amino acids such as valine, methionine, phenylalanine, and threonine during malnutrition (14, 15); thus, the above amino acid-related genes might be altered to increase the amino acid supply. Lipids and carbohydrates play important roles in providing energy for the organism (16, 17). In terms of lipid transport and metabolism, DEGs related to fatty acid synthesis (*FADS3*), fatty acid oxidation (*ACSL6*), triglyceride and cholesterol ester hydrolysis (*LIPA*), and phospholipid metabolism (*PLD3*, *PLD4*, and *LPCAT1*) were all downregulated in the TR group except for *HSDL2* (Fig. 5b). For carbohydrate transport, *MFSD2A*, *SVOPL*, and *SLC22A5* were upregulated while *SLC37A2*, *SLC13A1*, and *ADD2* were downregulated in the TR group. DEGs related to carbohydrate degradation, including *UGT2C1*, *UGT1A9*, *PYGM*, and *RGN*, were all downregulated in the TR group, except for *UGT1A1* (Fig. 5c). The inhibited lipid and carbohydrate degradation was probably caused by the accumulated VFAs in the cecum, since VFAs can provide available energy for the body (18). For energy production and conversion, a DEG related to energy expenditure (*PCK2*) was upregulated, while DEGs involved in energy production (*ALDH1A2* and *ALDH1A3*) were downregulated, except for *GSR* and *ALDH1L2* (Fig. 5d). Overall, amino acid synthesis and transport were enhanced while amino acid degradation and modification, peptidases, lipid metabolism, and carbohydrate degradation were inhibited in cecal epithelium of undernourished ewes; these findings might correlate with the accumulated VFAs and the changed energy production and conversion system.

### Undernutrition inhibited the PI3K signaling pathway in cecal epithelium.

**FIG 6:**
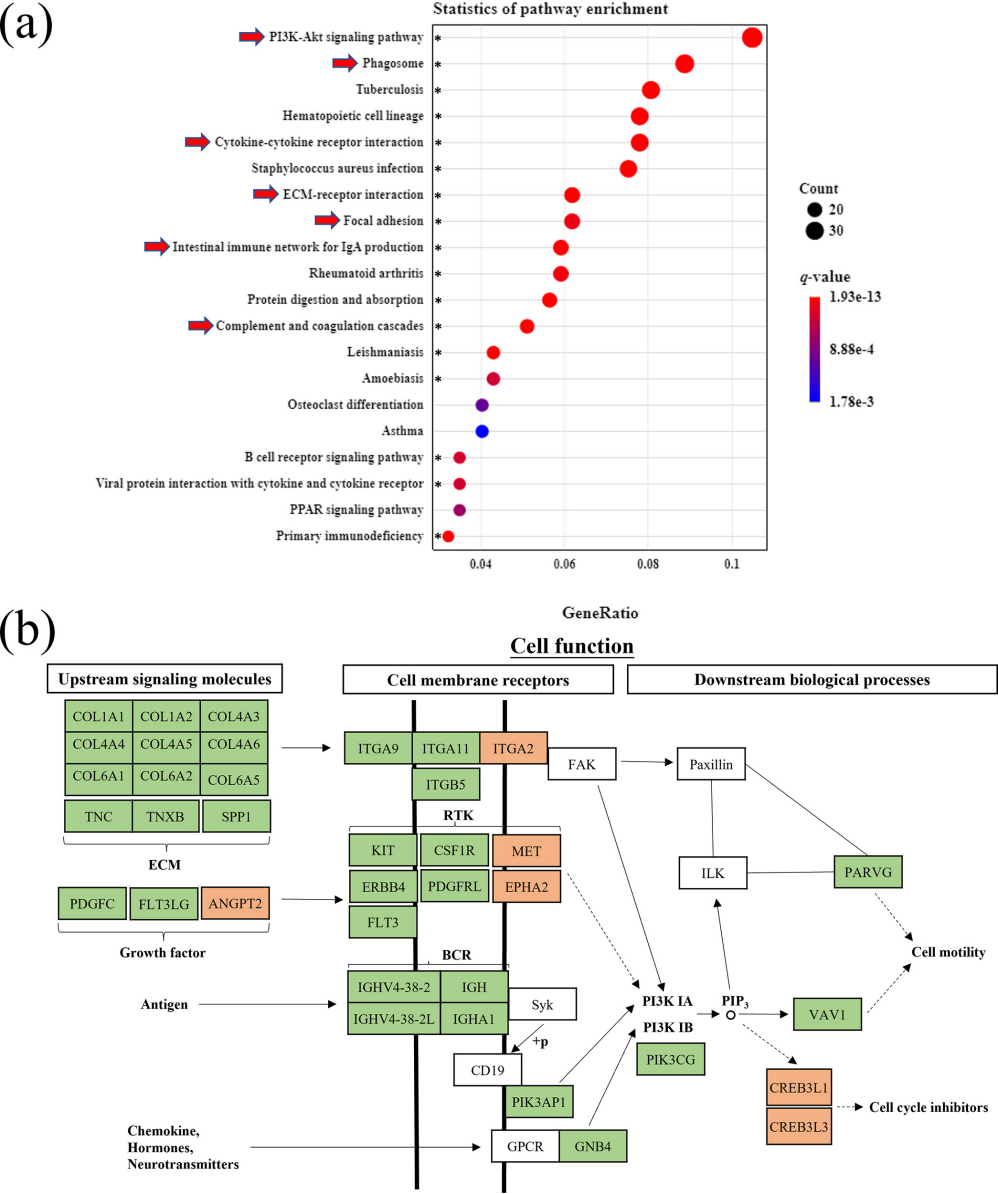
Undernutrition inhibited the proliferation and renewal of cecal epithelial cells through ECM-receptor interactions and the PI3K signaling pathway. (a) KEGG pathway enrichment analysis of DEGs. Asterisks indicate the significantly enriched pathways (*q *< 0.0005). (b) Schematic diagram of the signaling involved in the PI3K-Akt signaling pathway, ECM-receptor interaction, and focal adhesion. Red background indicates upregulated DEGs, while green background indicates downregulated DEGs. COL1A1, collagen type I alpha 1 chain; TNC, tenascin C; SPP1, secreted phosphoprotein 1; PDGFC, platelet-derived growth factor C; FLT3LG, FMS-related receptor tyrosine kinase 3 ligand; ANGPT2, angiopoietin 2; ITGA9, integrin subunit alpha 9; FAK, focal adhesion kinase; RTK, receptor tyrosine kinases; KIT, KIT proto-oncogene, receptor tyrosine kinase; CSF1R, colony-stimulating factor 1 receptor; MET, MET proto-oncogene, receptor tyrosine kinase; ERBB4, erb-b2 receptor tyrosine kinase 4; EPHA2, EPH receptor A2; BCR, B-cell receptor; IGH, immunoglobulin heavy locus; Syk, spleen tyrosine kinase; CD19, CD19 molecule; PIK3AP1, PI3K adaptor protein 1; GNB4, G protein subunit beta 4; PIK3CG, phosphatidylinositol-4,5-bisphosphate 3-kinase catalytic subunit gamma; PIP3, phosphatidylinositol-3,4,5-trisphosphate; ILK, integrin-linked kinase; PARVG, parvin gamma; VAV1, VAV guanine nucleotide exchange factor 1; CREB3L1, cyclic AMP-responsive element binding protein 3 like 1.

To further analyze the DEGs, we performed KEGG pathway enrichment analysis. As shown in Fig. 6a, the phosphoinositide 3 kinase-protein kinase B (PI3K-Akt) signaling pathway (*q *< 0.0001), phagosome (*q *< 0.0001), cytokine-cytokine receptor interaction (*q *< 0.0001), extracellular matrix (ECM)-receptor interaction (*q *< 0.0001), focal adhesion (*q *= 0.0002), intestinal immune network for immunoglobulin A (IgA) production (*q *< 0.0001), and complement and coagulation cascades (*q *< 0.0001) were enriched significantly by DEGs. ECM-receptor interactions, PI3K-Akt signaling pathway, and focal adhesion were tightly interconnected with each other and shared some DEGs; therefore, the DEGs enriched in these three pathways were combined and analyzed together (Fig. S5). As shown in Fig. 6b, ECM, growth factor, antigen, and chemokine interact with cell membrane receptors and then act on PI3K IA and PI3K IB to regulate cell motility and the cell cycle. Most DEGs involved in upstream signaling molecules for ECM (*COL1A1*, *COL1A2*, *COL4A3*, *COL4A4*, *COL4A5*, *COL4A6*, *COL6A1*, *COL6A2*, *COL6A5*, *TNC*, *TNXB*, and *SPP1*) and growth factor (*PDGFC* and *FLT3LG*), cell membrane receptors (*ITGA9*, *ITGA11*, *ITGB5*, *KIT*, *ERBB4*, *PDGFRL*, *CSF1R*, *FLT3*, *IGHV4-38-2*, *IGHV4-38-2L*, *IGH*, *IGHA1*, *PIK3AP1*, and *GNB4*), and downstream biological process cell motility (*PARVG* and *VAV1*) of the PI3K (*PIK3CG*) signaling pathway were downregulated in the TR group, except growth factor *ANGPT2*, cell membrane receptors *ITGA2*, *MET*, and *EPHA2*, and cell cycle inhibitors *CREB3L1* and *CREB3L3*. In summary, undernutrition repressed upstream ECM and growth factor genesis and their interactions with cell membrane receptors, thereby inhibiting the PI3K signaling pathway, and affected downstream cell motility, apoptosis, and metabolism in cecal epithelium.

### Undernutrition reduced immune function in cecal epithelium.

**FIG 7:**
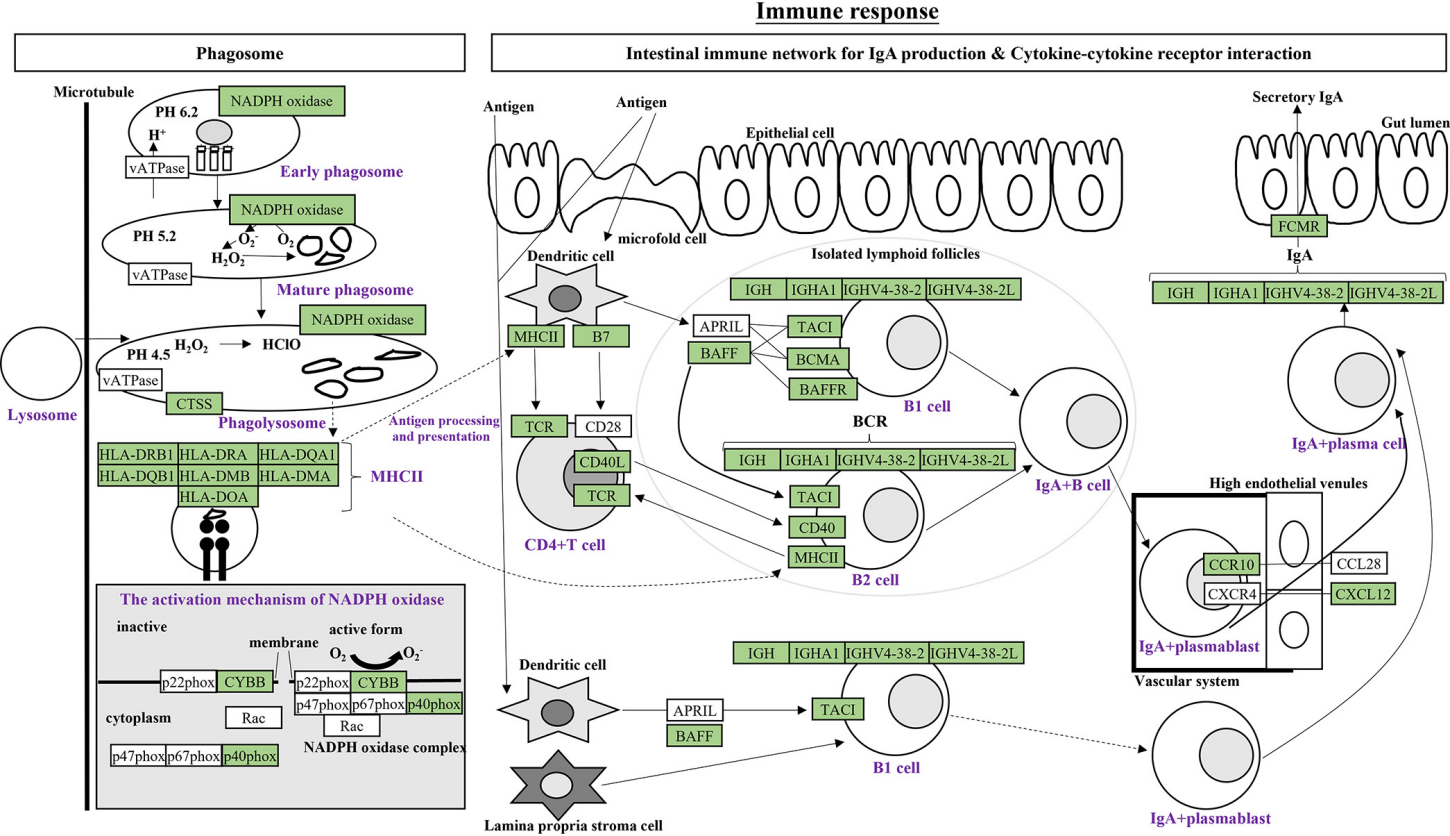
Undernutrition suppressed the intestinal immune function of cecal epithelium. Schematic diagram of the signaling involved in the phagosome, intestinal immune network for IgA production, and cytokine-cytokine receptor interaction. Green background indicates downregulated DEGs. NADPH, NADP oxidase; CTSS, cathepsin S; HLA, major histocompatibility complex, class II; CYBB, cytochrome B-245 beta chain; NCF4, neutrophil cytosolic factor 4; APRIL, a proliferation-inducing ligand; BAFF, B cell activating factor belonging to the tumor necrosis factor (TNF) family; TACI, transmembrane activator and calcium modulator and cyclophilin ligand interactor; GPA33, glycoprotein A33; TNFRSF13B, TNF receptor superfamily member 13B; CCR10, C-C motif chemokine receptor 10; CXCL12, C-X-C motif chemokine ligand 12; FCMR, Fc Mu receptor.

Phagosomes, the intestinal immune network for IgA production, and cytokine-cytokine receptor interactions are all involved in the immune response and share many DEGs, so we combined these DEGs for analysis (Fig. 7). In these three pathways, 55 DEGs were downregulated while only 10 DEGs were upregulated in the TR group (Fig. S6). Early phagosomes become mature phagosomes through membrane fusions and then bind to lysosomes to become phagolysosomes (19), in which NADPH oxidases play important roles (20). Phagolysosomes promote antigen degradation to form major histocompatibility complex II (MHC-II) (21), which then enters the intestinal immune network to participate in antigen presentation and processing. Dendritic cells interact with B cells and T cells to promote B1 and B2 cell differentiation into IgA plasma blast cells and then into IgA plasma cells, which produce IgA and then form secretory IgA (22). Our results showed that DEGs associated with phagolysosome formation (*CYBB* of NADPH oxidase, *NCF4* of p40phox, and *CTSS*), MHC-II antigen presentation and processing (*HLA-DRB1*, *HLA-DRA*, *HLA-DQA1*, *HLA-DQB1*, *HLA-DMB*, *HLA-DMA*, and *HLA-DOA*), B1 and B2 cells differentiation (*TNFSF13B* of BAFF, *TNFRSF13B* of TACI, *TNFRSF17* of BCMA, *TNFRSF13C* of BAFFR, *GPA33* of TCR, *CD86* of B7, *CD40*, *CD40LG* of CD40L, and *IGHV4-38-2*, *IGHV4-38-2L*, *IGH*, and *IGHA1* of BCR), and IgA production and secretion (*CCR10*, *CXCL12*, and *FCMR*) were all downregulated in the TR group. These findings implied that undernutrition during late gestation inhibited phagolysosome maturation, antigen presentation and processing, B cell differentiation, and antibody IgA production and secretion in cecal epithelium, which ultimately suppressed the intestinal immune network.

**FIG 8:**
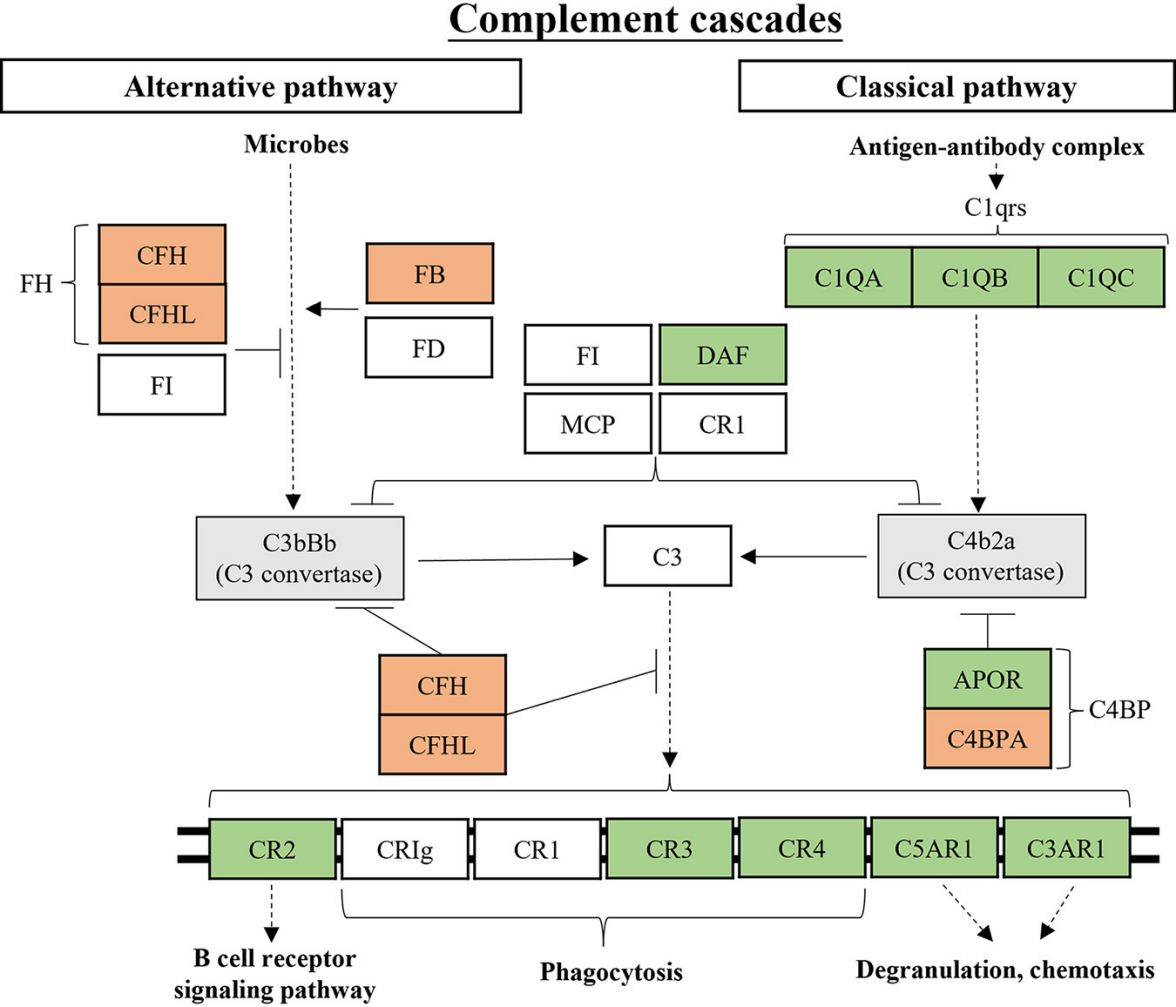
Undernutrition inhibited the inflammatory response of the cecal epithelium as shown in this schematic diagram of signaling involved in the complement cascade pathway. Red background indicates upregulated DEGs, while green background indicates downregulated DEGs. CFH, complement factor H; C1QA, complement C1q A chain; DAF, decay acceleration factor; MCP, membrane cofactor protein; CR1, complement C3b/C4b receptor 1; APOR, apolipoprotein R; C4BPA, complement component 4 binding protein alpha; C5AR1, complement C5a receptor 1.

The expression of DEGs enriched in complement and coagulation cascades were shown in a heat map (Fig. S7). As shown in Fig. 8, the alternative pathway is activated by microbes to form C3bBb complex (23) and the classical pathway is activated to form C4b2a complex when antigen-antibody complex binds to C1qrs (24). Our results demonstrated, for DEGs related to the alternative and classic pathways, *C1QA*, *C1QB* and *C1QC* of C1qrs, *CD55* of DAF, and *APOR* of C4BP were downregulated, while *CFH* and *CFHL* of FH, *CFB* of FB, and *C4BPA* of C4BP were upregulated in the TR group. Ultimately, both the alternative and classical pathways produced C3 convertases that can affect immune-related genes through a series of reactions. Among these DEGs, *CR2* is associated with the B-cell receptor signaling pathway and enhances humoral immune responses (25), CR3 and CR4 are associated with phagocytosis and play important roles in innate and adaptive immunity (26), while *C3AR1* and *C5AR1* work in degranulation and chemotaxis (27). *CR2*, *C3AR1*, *C5AR1*, and *ITGB2* of CR3 and CR4 were downregulated in the TR group.

## DISCUSSION

In this study, we investigated the effects of undernutrition during late gestation on microbiota in cecal digesta and substance metabolism, signal transduction, and immune functions in the cecal epithelium and their interaction. Our results showed that the concentrations of acetate, propionate, isobutyrate, butyrate, isovalerate, valerate, total VFAs, and microbial proteins in the cecal digesta were all increased upon undernutrition, which were similar to the results of increased concentrations of propionate, isobutyrate, butyrate, isovalerate, and valerate in the colonic digesta of pregnant ewes in the presence of malnutrition (6). As we know, the low flow rate of intestinal digesta increases digestion time and changes microbial communities (28). The increased VFAs and microbial protein content in the hindgut upon undernutrition are probably caused by the decreased flow rate of sizeable intestinal digest due to the low feed intake (6). Further, we found the molar proportions of acetate, butyrate, and valerate in the cecal digesta were significantly changed upon undernutrition. The molar proportion of acetate decreased in spite of the increased concentration of acetate, which was caused by the relatively lower increasing rate of acetate concentration compared to total VFA concentration. Essentially, the changed molar proportions of VFAs resulted from the altered microbial community structures (29). In previous studies, *Clostridia* vadinBB60 group_norank is negatively correlated with butyrate proportion (6); *Oscillospiraceae*_uncultured can produce butyrate from their fermentable carbon source (10, 30); *Peptococcaceae* is positively correlated with butyrate concentration (31); *Peptococcaceae*_uncultured is positively correlated with valerate proportion (32); *Ruminococcus* is essential for carbohydrate-active enzymes that can produce acetate from maltose oligosaccharides and transient glucose produced during starch degradation (33); and *Rikenellaceae* dgA-11 gut group is positively correlated with acetate concentration (34) and *Rikenellaceae* RC9 gut group is positively correlated with acetate proportion (35). In the current study, the correlations between altered microbial genera and VFA proportions were highly consistent with those described in the references mentioned above. Thus, it is probable that the increased butyrate proportion was caused by the increased relative abundances of *Oscillospiraceae*_uncultured and *Peptococcaceae*_uncultured and the decreased relative abundance of *Clostridia* vadinBB60 group_norank; the increased valerate proportion was caused by the increased relative abundance of *Peptococcaceae*_uncultured; and the decreased acetate proportion was caused by the decreased relative abundances of *Ruminococcus*, *Rikenellaceae* dgA-11 gut group and *Rikenellaceae* RC9 gut group. Taken together, these findings indicated that undernutrition during late gestation affected microbial community in the cecal digesta and then changed the molar proportions of VFAs. However, the slow flow rate of hindgut digesta due to low feed intake increased the accumulation of VFAs and microbial protein concentrations.

Nutritional intake affects the composition of the gut microbial community and the physiological metabolism of the host (36). Both lipid and carbohydrate metabolism (37, 38) and VFAs (10) can provide energy, while amino acid synthesis requires energy (39). Lipid and carbohydrate metabolism are enhanced in ruminal epithelium to alleviate the energy deficiency when VFA concentrations are reduced upon undernutrition (7). In the current study, the high concentrations of VFAs produced by microbiota in cecal digest probably promoted amino acid synthesis but repressed amino acid, carbohydrate, and lipid degradation in the epithelium.

The altered substance metabolism induced by malnutrition affects epithelial cell proliferation and apoptosis, thus affecting epithelial tissue morphology, but the specific signaling regulatory mechanisms are not clear. ECM plays an important role in the regulation of cellular phenotype and behavior through ECM-cell interactions based on ECM receptors on the cell membrane (40). ECM-cell interactions mediate cell adhesion and cell signaling and then regulate cell proliferation, differentiation, migration, and apoptosis (40). In the current study, most of the DEGs related to ECM and ECM-receptor interactions were downregulated in the cecal epithelium upon undernutrition, which might result from the inhibition of biological processes in the microbiota (Fig. 3d). ECM can induce FAK activation and promote PI3K phosphorylation (40). The PI3K signaling pathway plays an important role in cell proliferation, differentiation, and apoptosis (41). In the present study, it was found that PI3K and its downstream DEGs involved in cell motility were downregulated while DEGs related to cell cycle inhibition were upregulated (Fig. 6b). It has been reported that DNA replication and cell cycle were extremely inhibited due to the inhibition of ECM-receptor interactions caused by inactivation of the JAK3-STAT2 signaling pathway in rumen epithelium of undernourished ewes (7). In the present study, the repressed ECM-receptor interaction, PI3K signaling pathway, and cell motility and cell cycle in cecal epithelium of undernourished ewes were highly similar to this. Taken together, undernutrition during late gestation repressed ECM genesis and ECM-receptor interaction, which further inhibited the PI3K signaling pathway and its downstream cell motility and cell cycle. This probably affected cell proliferation and apoptosis for epithelium update and further changed epithelial morphology and cecal weight.

It is well known that malnutrition is the most prevalent cause of immunodeficiency worldwide, and abnormalities in the immune system affect both innate and adaptive immunity (42). Four immune-related pathways were identified in the KEGG enrichment analysis of DEGs in the present study. Phagosomes are critical for killing and subsequent degradation of phagocytosed microorganisms by fusing with lysosomes to form phagocytic lysosomes, and they play an important role in the long-term antigen presentation of MHC-II (19). Malnourished patients do not release acid phosphatase from lysosomes during phagocytosis, which can inhibit phagocytic lysosome formation (43). In our results, NADPH oxidase was inhibited, which would affect phagocytosis in lysosomes as well as MHC-II formation and antigen delivery. B cells differentiate into plasma cells and produce secretory IgA, which is the main specific immune antiviral and antimicrobial defense method on the mucosal surface of the gastrointestinal tract (44). A decrease in T cells and IgA-containing B cells in the intestinal mucosa of severely malnourished children has been reported, as well as a decrease in secretory IgA production by the plasma cells they form (44). Our results suggested that the process of secretory IgA production by plasma cells generated from T cells and IgA-containing B cells is inhibited in the cecal epithelium of malnourished ewes. The complement cascade is a key actor of the innate immune system that contributes to the rapid clearance of pathogens and dead or dying cells and has the potential to influence the inflammatory immune response (45). Protein-energy malnutrition can alter the total complement system in a mouse model (46). In this study, both the alternative and classic pathways in the complement system were repressed in cecal epithelium upon malnutrition. Collectively, undernutrition during late gestation inhibited phagolysosome formation, antigen delivery and processing, T cell and B cell differentiation, secretory IgA production, and the complement system in cecal epithelium of ewes.

**FIG 9:**
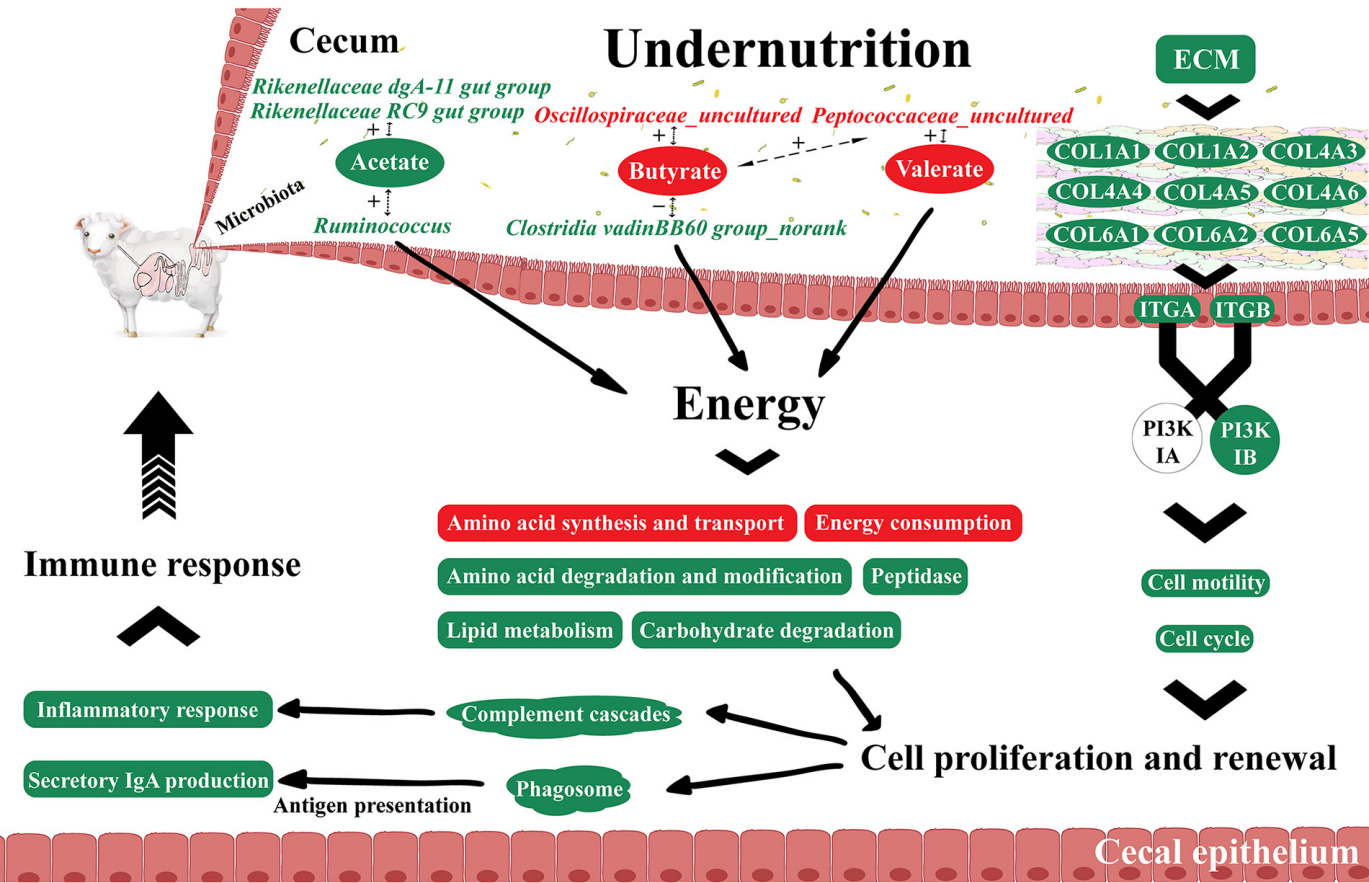
Comprehensive response of cecal microbiota and epithelium to undernutrition and the interactions between them. +, positive correlation; −, negative correlation. Red or green background and font represent upregulated or downregulated microbiota, genes, metabolites, and biological processes in undernourished ewes, respectively.

Undernutrition during late gestation affected the relative abundances of microorganisms involved in VFA production and change the molar proportions of VFAs (Fig. 9). However, the low flow rate of intestinal digesta caused by low feed intake resulted in the accumulation of VFAs and microbial proteins in cecal digesta, which altered the energy regimen and therefore affected the substance metabolism in cecal epithelium. ECM and ECM-receptor interactions were inhibited, which repressed cell motility and cell cycle via the PI3K signaling pathway and affected cecal epithelial morphology and cecal weight. These changes finally lowered the immune response function in cecal epithelium.

## MATERIALS AND METHODS

### Animal experimental design and sample collection.

This study is from a large project which used 16 pregnant ewes to explore the effects of malnutrition during late gestation on maternal and fetal metabolism. The specific design and operation of this animal experiment has been described in detail (13). In simple terms, 16 ewes (pregnancy for 108 days with 2 to 4 fetuses, weights of 60.6 ± 4.9 kg) were allowed to feed freely for 7 days to calculate the feed intake. They were then equally divided into the CON and TR groups on 115 days of gestation, and ewes in the CON and TR groups were fed 100% and 30% of the *ad libitum* feeding level, respectively, for 15 days to establish an undernourished sheep model. The diet formula and nutrient compositions were detailed in our previous articles (7). The crude protein content and metabolic energy in the total mixed ratio were 14.71% and 11.64 MJ/kg, respectively, which met the protein and energy requirements for ewes carrying multiple fetuses based on the calculated feed intake. The ewes were slaughtered at 4 h after the morning feeding on 130 days of gestation when undernourished sheep model was successfully established, and cecal epithelium samples and cecal digesta samples were collected immediately and stored in liquid nitrogen.

**Ethics statement.** The experimental design and procedures for this study were approved by the Animal Care and Use Committee of Nanjing Agricultural University [SYXK(Su)2015-0656].

### Determination of cecal fermentation parameters.

pH of cecal digesta was immediately detected after slaughter using a pH meter (47). VFAs in cecal digesta were detected using a gas chromatograph and the method described by Qin (48). In detail, cecal digesta were diluted with distilled water (1:5), and the mixture was centrifuged at 2,000 × *g* for 10 min. The supernatant was mixed with 25% (wt/vol) metaphosphoric acid to measure the VFA concentrations. The gas chromatograph used a capillary suction column (30 m by 0.32 mm by 0.25 μm) with a column temperature of 130°C, a vaporization temperature of 180°C, and a hydrogen ion flame detector with a detection temperature of 180°C (49). Microbial protein content of cecal digesta was measured by the Comas Brilliant Blue method (50). The molar proportions of VFAs were calculated using the ratio of target VFA concentration to total VFA concentration.

### Cecal weight and morphological changes.

Digesta in the cecum were removed immediately after slaughter to measure the emptied cecum tissue weight. A piece of cecal sample was fixed in 4% paraformaldehyde for 24 h and embedded in paraffin to make hematoxylin and eosin-stained sections for morphological observation (51).

### 16S rRNA gene sequencing analysis of microorganisms in cecal digesta.

DNA of microorganisms in the cecal digesta was extracted using the cetyltrimethylammonium bromide method (52). The V3-V4 region of the 16S rRNA gene was subsequently amplified using the 16S rRNA universal gene primers 341F (5′- CCTAYGGGRBGCASCAG -3′) and 806R (5′-GGACTACNNGGGTATCTAAT -3′) (9). The amplicons obtained were purified using the AxyPrep DNA gel extraction kit (Axygen Biosciences, Union City, CA), and the purified PCR products were used to construct sequencing libraries using the Illumina TruSeq DNA sample preparation kit (Illumina, San Diego, CA). Cluster generation, template hybridization, isothermal amplification, linearization, closure, denaturation, and primer hybridization were performed using the Illumina TruSeq PE cluster kit (V2; Illumina, San Diego, CA, USA) and TruSeq SBS kit (V5; Illumina, San Diego, CA, USA). The entire library was sequenced on the Illumina MiSeq platform according to protocol standards (53). QIIME (v.1.9.0) was used to process the raw data, UPARSE (v.7.1) was used to cluster OTUs based on a 97% sequence similarity level, and UCHIME was used to identify chimeric sequences. The taxonomic analysis on the OTU representative sequences was performed using RDP classifier (v. 2.2) and compared to the SILVA database (v. 132). Rarefaction curves were used to assess the sequencing depth, and alpha diversity indexes were used to estimate bacterial diversity. We also performed PCoA based on Bray-Curtis, unweighted UniFrac, and weighted UniFrac metrics to assess the differences of cecal microbiota between the two groups.

### Transcriptome assay of cecal epithelium.

Total RNA was extracted from cecal epithelium using the TRIzol method (54) and checked for RNA concentration, purity, and integrity (55). Ten total RNA samples (5 per group) were then randomly selected to prepare cDNA libraries. The mRNA was purified from total RNA using the magnetic bead method, then fragmented and reverse transcribed into cDNA. Purified cDNA was ligated to sequencing adapters and amplified using the NEBNext Ultra RNA library preparation kit. Fragments with appropriate length were selected to obtain a cDNA library and sequenced on an Illumina Hi-Seq 2500 platform (Biomarker, Beijing, China). Raw data were screened to generate high-quality reads, which were then mapped to the sheep reference genome 4.0 using HiSAT2 (56, 57). The fragments per kilobase of transcript per million fragments mapped (FPKM) values were calculated to show the expression of all genes. DEGs were screened with the criteria of a false-discovery rate (FDR) of  <0.05 and fold change (FC) of >1.5 or <0.667 using DESeq2 software (v1.6.3). The Biomarker platform (www.biocloud.net) was used to plot the volcano map and clustering heat map and to perform COG functional classification and KEGG pathway enrichment analysis.

### Real-time quantitative PCR analysis of genes in cecal epithelium.

A QuantStudio 5 real-time PCR instrument (Applied Biosystems, Foster, CA, USA) was used to quantify the expression of target genes. The PCR mixture was a 20-μL reaction system containing 2.0 μL of cDNA template, 10 μL of SYBR Premix Ex Taq, 0.4 μL of forward primer (10 μM), 0.4 μL of reverse primer (10 μM), 0.4 μL of ROX reference dye, and 6.8 μL of sterilized distilled water. The PCR amplification procedure was as follows: predenaturation at 95°C for 30 s, 40 cycles of 95°C for 5 s and 60°C for 34 s, and a melting curve program (60°C to 99°C with a ramp rate of 0.1°C/s and the measurement of fluorescence). The expressional levels of target genes were calculated according to the threshold cycle (2^−ΔΔ^*^CT^*) method using glyceraldehyde 3-phosphate dehydrogenase as the housekeeping gene. Primers of the genes for the real-time quantitative PCR are shown in Table S1.

### Statistical analysis.

Independent-sample *t* test was performed in IBM SPSS Statistics 25 (SPSS Inc., Chicago, IL, USA) to assess the significance of differences of cecal weight, cecal fermentation parameters, and gene expressional levels between the two groups. Microbial alpha diversity and relative abundances of bacteria at the phylum and genus levels were then tested using nonparametric tests in IBM SPSS Statistics 25. Data as shown as means ± standard errors of the means, and *P* values of <0.05 indicated significant difference. Spearman correlation coefficients (*r*) and significance tests between microbiota and fermentation parameters were calculated in IBM SPSS Statistics 25 using bivariate correlations (*n* = 16), where a *P* value of <0.05 indicated a significant correlation. The *P* value from the KEGG enrichment analysis was calculated using the clusterProfiler in the R package and corrected for multiple hypothesis testing to yield a *q* value (58).

### Data availability.

Raw reads of transcriptome sequencing of cecal epithelium are available at GEO. under accession number GSE221270. Raw reads of 16S rRNA gene sequencing are available at the National Center for Biotechnology Information Sequence Read Archive (SRA), under SRA project number PRJNA914498.
