# Supplementary material for: Undernutrition Disrupts Cecal Microbiota and Epithelium Interactions, Epithelial Metabolism, and Immune Responses in a Pregnant Sheep Model
Source: Microbiol Spectr. 2023 Mar 28;11(2):e05320-22. doi: 10.1128/spectrum.05320-22 (PMC10100782; doi:10.1128/spectrum.05320-22)
Supplement: Supplemental file 1 — Supplemental material. Download spectrum.05320-22-s0001.pdf, PDF file, 0.7 MB [file spectrum.05320-22-s0001.pdf]

Table S1 Gene primers used for real-time quantitative PCR.

Gene symbol	Primer sequence	Accession number	Length (bp)
ASNS	F: CCTTTTTCGCCAGGACATTA<br>R: TGATAAAAGGCAGCCAATCC	XM_012176371.4	240
PSPH	F: AACAGGTGCAGAGGCTCCTA<br>R: TGAGCTTTGAAGCAACATGC	XM_012103911.4	150
SLC7A5	F: CTCTGCGTGCTGCTACTCAC<br>R: CTTGGGATCCAGATTGGCTA	XM_027977720.2	162
PCK2	F: GCTACAACTTTGGGCGCTAC<br>R: GTCGGCAGATCCAGTCTAGC	XM_015096868.3	168
COL1A1	F: CGTGATCTGCGACGAACTTA<br>R: TCCAGGAAGTCCAGGTTGTC	XM_027974705.2	211
FCMR	F: GACCGAGTCTCCTCAAGCAC<br>R: GGTTGGAGCCTCTCTGTCTG	XM_027976242.2	215
UGT1A9	F: AGGAATATGCAATGGCGTTC<br>R: CACCCAGAATACAGCCAGGT	NM_001009189.1	247
CXCL12	F: CGTCAAGCACCTCAAGATCC<br>R: GTTCCCAGGGCAGAGGTC	XM_027962332.2	180

TNFRSF17	F: TTGGGCCTGAGCTTGATAGT<br>R: CCAGGCCTCTCAAAAGAAGA	XM_015104084.3	190
PIK3CG	F: ACTGTCACCCCATAGCCTTG<br>R: TTTCTTGCTGTCCCCATTTC	XM_004007845.5	240
PYGM	F: CGCATCGGAGAGGAATACAT<br>R: AGTTGAGGAGCTGGCGTTTA	NM_001009192.2	223
ITGB5	F: CCCGGTGACAAGACTACGTT<br>R: GAAGTTGCTGGTGAGCTTCC	XM_027956587.2	168
FADS3	F: ACTTGCCCTACAACCACCAG<br>R: GTGATCCACACGAACCAGTG	XM_027959463.2	247
SLC37A2	F: GGTGATCCAGCCCCTCAATA<br>R: ATGCCACTGATGAACATGCC	XM_012101850.4	153
CTSS	F: AATGGCACAAGTGCACAGAG<br>R: ATTCCCATTGAATGCTCCAG	XM_004002463.5	168
LIPA	F: ACACCTGGTCTCGGAAACAC<br>R: GCCTTGAGAATGACCCACAT	XM_012102434.3	161

Fig. S1

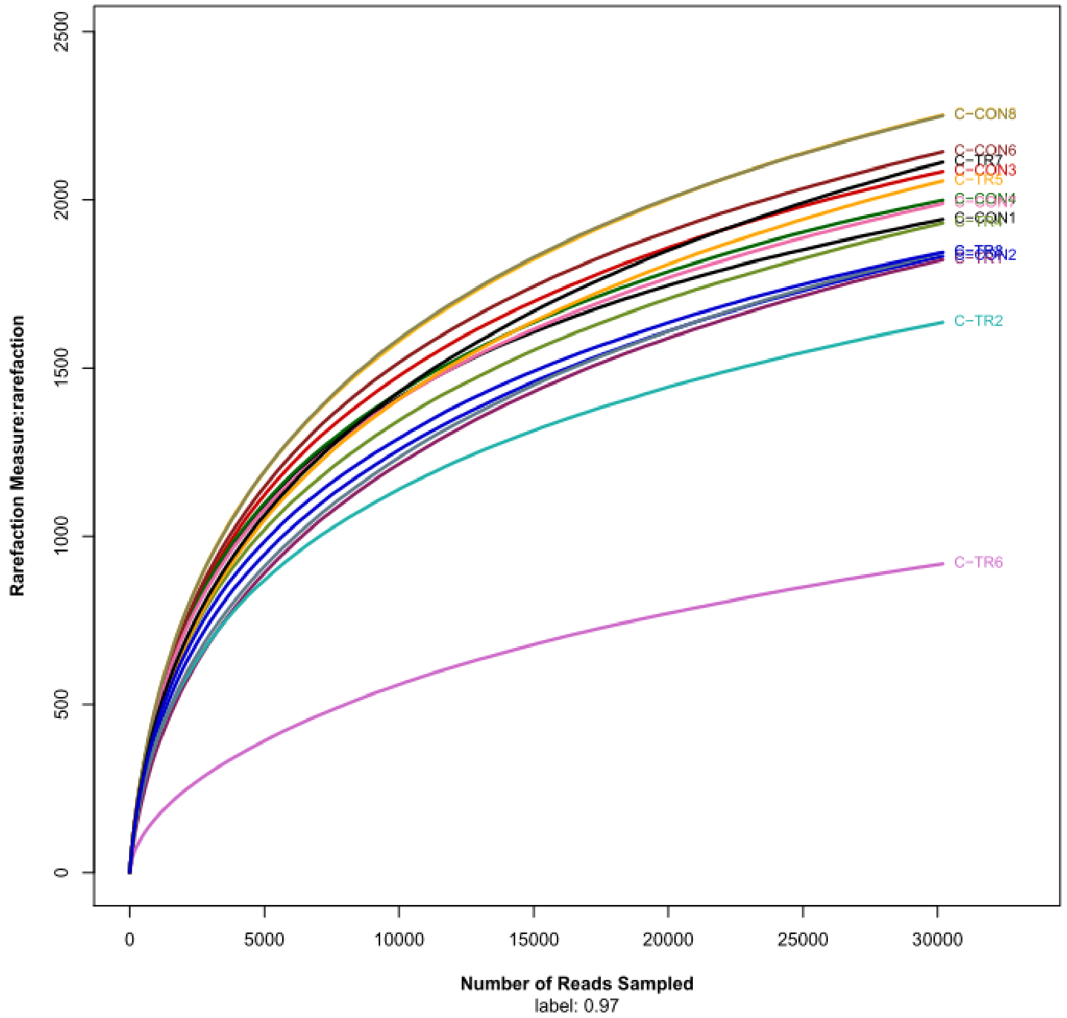

Fig. S1 Rarefaction curves based on OTUs for all the samples.

Fig. S2

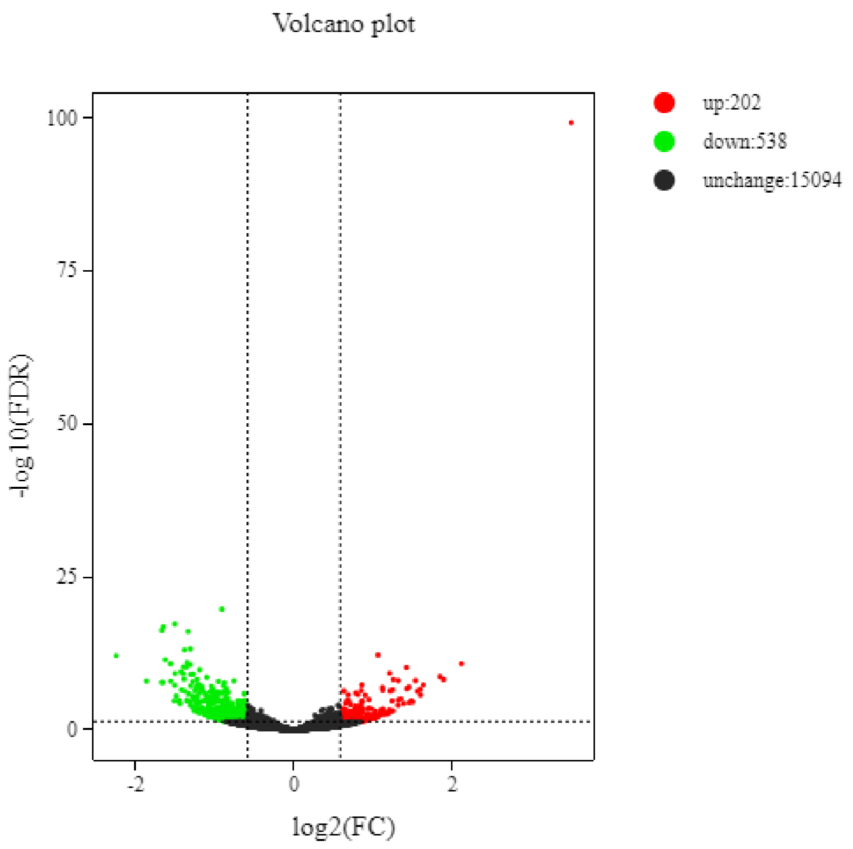

Fig. S2 Volcano plot of total genes from transcriptome sequencing of cecal epithelium. The screening criteria for DEGs were FDR < 0.05 and FC > 2 or < 0.667.

Fig. S3

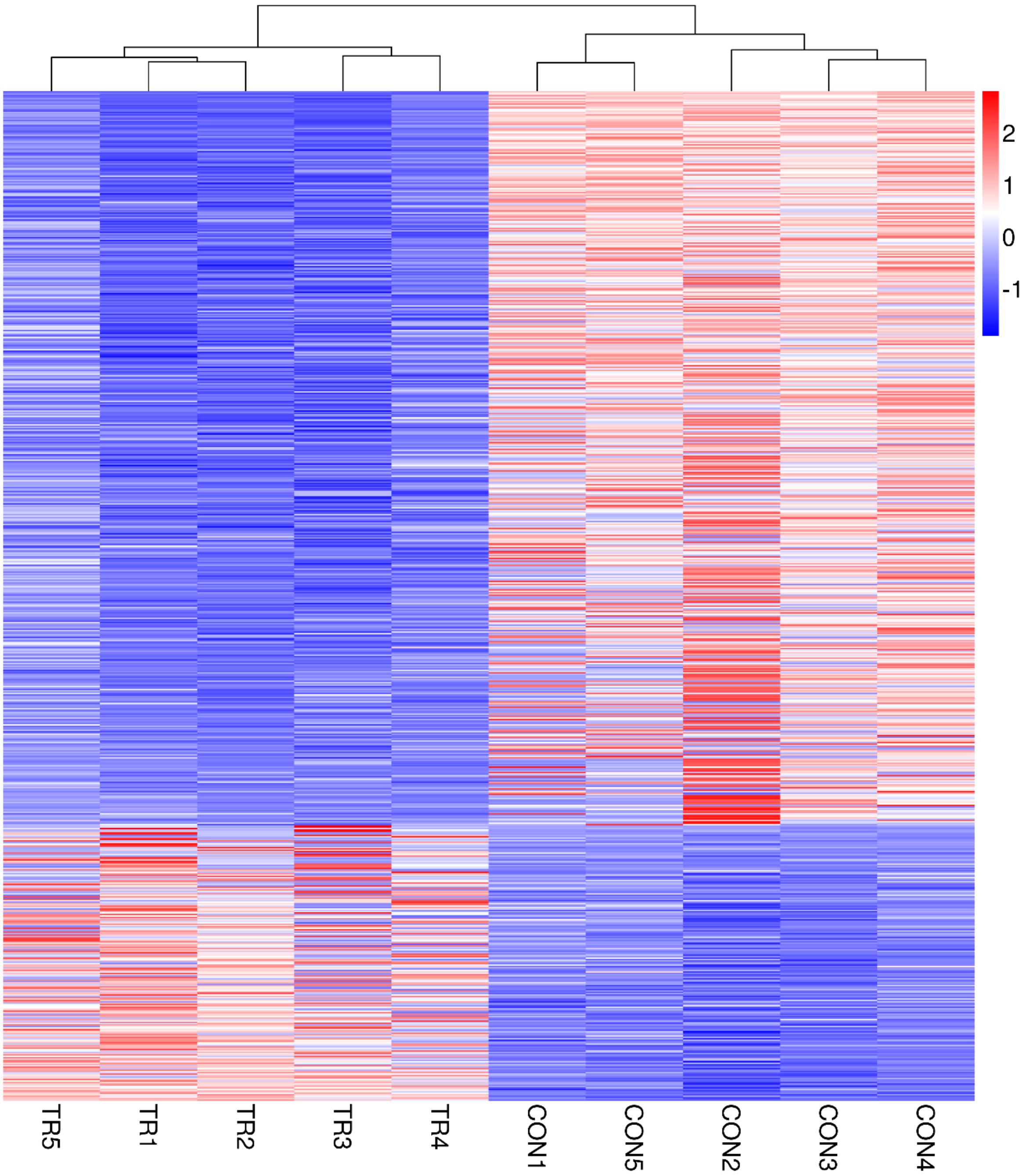

Fig. S3 Heat map drawn using DEGs by column clustering analysis in the cecal epithelium.

Fig. S4

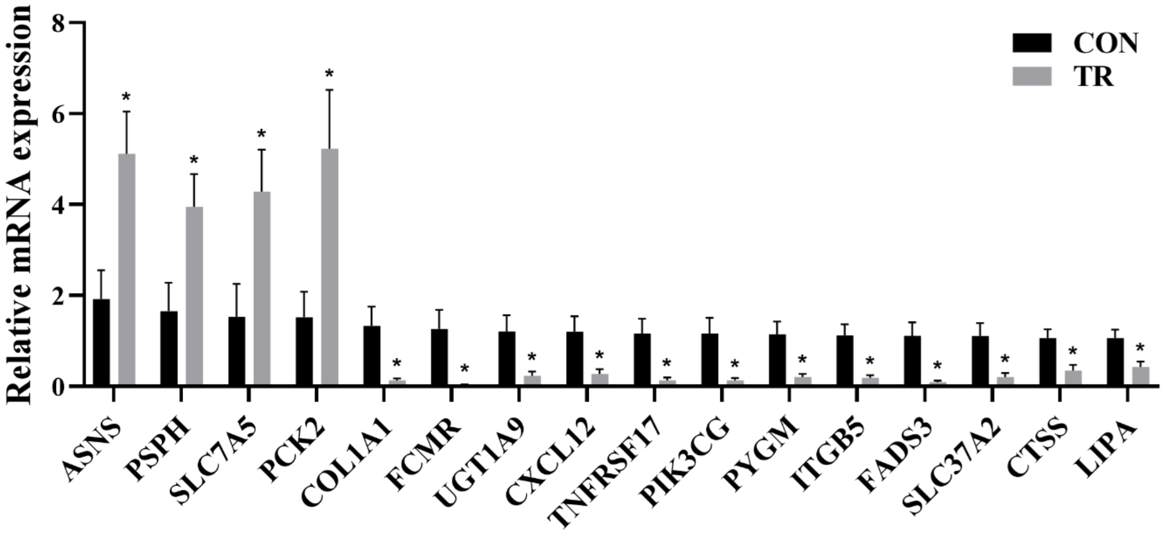

Fig. S4 Validation of RNA-sequencing data using real-time quantitative PCR. Data were represented as mean ± SEM. Asterisks indicate significant differences ($P < 0.05$).

Fig. S5

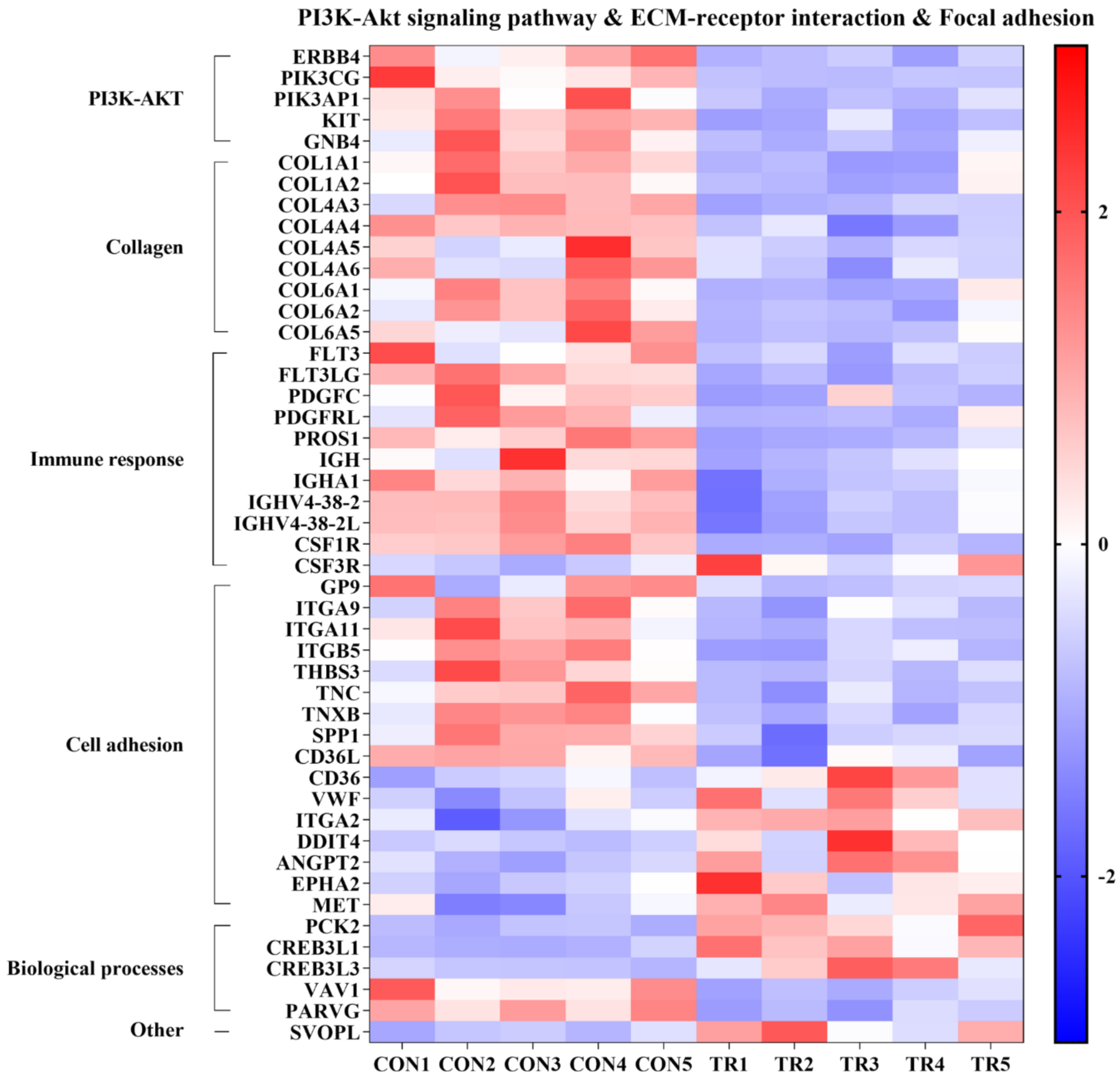

Fig. S5 Heat map of DEGs associated with PI3K-Akt signaling pathway, ECM-receptor interaction, and focal adhesion.

Fig. S6

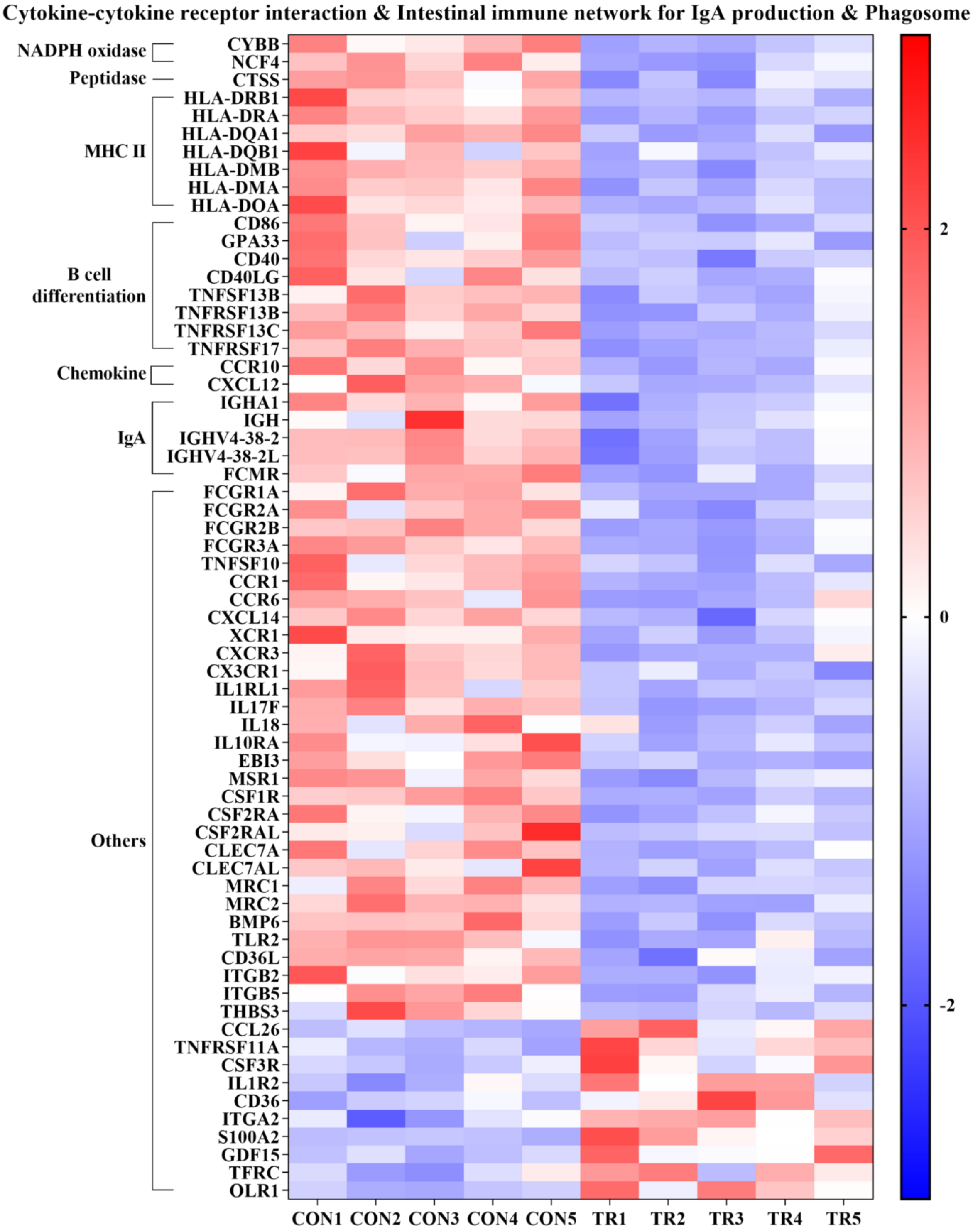
Cytokine-cytokine receptor interaction & Intestinal immune network for IgA production & Phagosome
NADPH oxidase
Peptidase
MHC II
B cell differentiation
Chemokine
IgA
Others
CYBB
NCF4
CTSS
HLA-DRB1
HLA-DRA
HLA-DQA1
HLA-DQB1
HLA-DMB
HLA-DMA
HLA-DOA
CD86
GPA33
CD40
CD40LG
TNFSF13B
TNFRSF13B
TNFRSF13C
TNFRSF17
CCR10
CXCL12
IGHA1
IGH
IGHV4-38-2
IGHV4-38-2L
FCMR
FCGR1A
FCGR2A
FCGR2B
FCGR3A
TNFSF10
CCR1
CCR6
CXCL14
XCR1
CXCR3
CX3CR1
IL1RL1
IL17F
IL18
IL10RA
EBI3
MSR1
CSF1R
CSF2RA
CSF2RAL
CLEC7A
CLEC7AL
MRC1
MRC2
BMP6
TLR2
CD36L
ITGB2
ITGB5
THBS3
CCL26
TNFRSF11A
CSF3R
IL1R2
CD36
ITGA2
S100A2
GDF15
TFRC
OLR1
CON1
CON2
CON3
CON4
CON5
TR1
TR2
TR3
TR4
TR5
2
0
-2

Fig. S6 Heat map of DEGs associated with phagosome, intestinal immune network for IgA production, and cytokine-cytokine receptor interaction.

Fig. S7

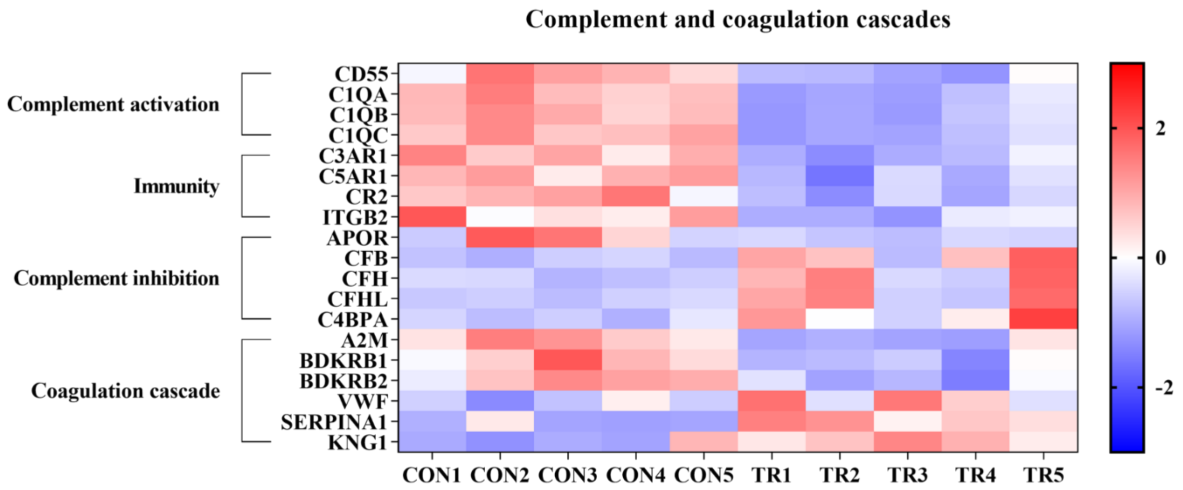

Fig. S7 Heat map of DEGs associated with complement and coagulation cascade.
